# Hypermetabolic Calcified Lymph Nodes on 18Fludeoxyglucose-Positron Emission Tomography/Computed Tomography in a Case of Treated Ovarian Cancer Recurrence: Residual Disease or Benign Formation?

**DOI:** 10.4274/mirt.22932

**Published:** 2016-06-06

**Authors:** Alexandra Nikaki, Athanasios Alexopoulos, Fani Vlachou, Vasiliki Filippi, Ioannis Andreou, Vasiliki Rapti, Konstantinos Gogos, Konstantinos Dalianis, Roxani Efthymiadou, Vassilios Prassopoulos

**Affiliations:** 1 SA Hygeia Hospital, Clinic of Nuclear Medicine and PET/CT, Athens, Greece; 2 SA Hygeia Hospital, Clinic of Pathology and Oncology, Athens, Greece; 3 SA Hygeia Hospital, Clinic of Radiology and PET/CT, Athens, Greece

**Keywords:** 18F-fludeoxyglucose, ovarian cancer, lymph nodes

## Abstract

The contribution of positron emission tomography/computed tomography (PET/CT) with 18F-fludeoxyglucose (FDG) in evaluating ovarian cancer recurrence even after a prolonged disease-free interval, and in therapy response is well-described. Calcifications observed in CT, although usually attributed to benign conditions, may actually represent active disease. Such an example of calcified formations is psammoma bodies. We present a case of 56-y. o. patient with ovarian cancer relapse at the supraclavicular area 18 years after complete response and disease-free interval. The patient received chemotherapy and underwent 18F-FDG-PET/CT for the evaluation of treatment response. Both CT corrected and uncorrected PET images showed hypermetabolism in the massively calcified lymph nodes in the neck, mediastinum, axilla and abdomen, indicative of active residual disease.

## Abstract (tr)

18Florodeoksiglikoz-pozitron emisyon tomografisi/bilgisayarlı tomografinin (18F-FDG PET/BT) over kanserinde tedavi yanıtını ve uzun bir hastalıksız aradan sonra nüksü değerlendirmede katkısı ortaya konulmuştur. Genellikle benign durumlara atfedilmesine rağmen BT’de gözlenen kalsifikasyonları aslında aktif hastalık bulgusu olabilir. Buna benzer kalsifiye oluşumlara bir örnek psammoma cisimleridir. Biz 56 yaşında, tedaviye tam yanıt ve 18 yıllık hastalıksız sağkalım sonrası supraklaviküler alanda nüks ile saptanan bir nüks over kanseri hastasını sunduk. Hasta kemoterapi aldı ve tedaviye cevabın değerlendirilmesi için 18F-FDG-PET/BT uygulandı. Hem düzeltilmiş hem düzeltilmemiş PET-BT görüntülerinde aktif rezidüel hastalığın göstergesi olarak boyun, mediastinum, koltuk altı ve karında kitlesel kalsifiye lenf düğümlerinde hipermetabolizma saptandı.

## INTRODUCTION

About 30-50/100.000 women per year are diagnosed with ovarian cancer and half of the deaths related to female genital system malignancies are attributed to ovarian cancer (1). The most common histologic type of ovarian cancer is epithelial, and the most common subtype of epithelial cancer is serous adenocarcinoma. More indolent forms also exist that are no longer considered as malignant (2). The presence of microcalcifications in the primary tumor and although less common, in metastatic lesions, have been described in the literature (3,4). In cases of serous papillary cancer, the calcifications can be related to psammoma bodies’ deposits (3,4). Calcification deposits depicted at computed tomography (CT) are more often attributed to benign conditions. Moreover, calcifications in the already identified malignant lymph nodes are considered as response to chemo and/or radiotherapy (5,6,7). However, calcified lymph nodes, even in the presence of extensive calcification, may be active and should not be ignored (3). The role of fludeoxyglucose-positron emission tomography/computed tomography (FDG-PET/CT) in restaging, evaluating therapy response in ovarian cancer, as well as its impact on management decisions for the patients is well-described and has been reviewed (1,8). Attenuation uncorrected images should be reviewed in order to avoid misinterpretation of calcifications. This case presentation demonstrates the utility of FDG-PET/CT in the evaluation of residual disease in large calcified lymph nodes in a patient who underwent chemotherapy for recurrent serous adenocarcinoma of the ovaries, 18 years after the initial diagnosis.

## CASE REPORT

**Figure 1:**
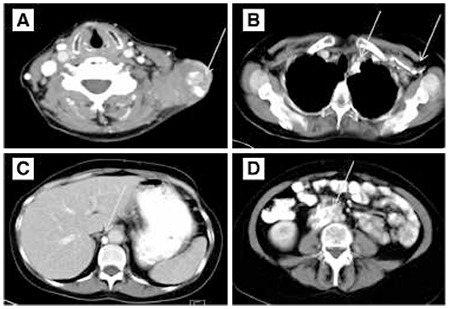
Enlarged calcified lymph nodes at the neck and the left supraclavicular region (A), the left axilla (B), the mediastinum (B), the retrocrural space on the right (C) and the paraaortic area (D) (arrows)

**Figure 2:**
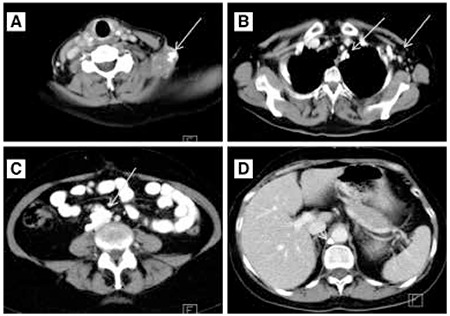
After 2 cycles of chemotherapy, the patient underwent computed tomography for evaluation of treatment response. Size reduction and presence of further calcification in the described lymph nodes were noted. Some of the lymph nodes were completely replaced by calcium deposits (A, B, C, D) (arrows)

**Figure 3:**
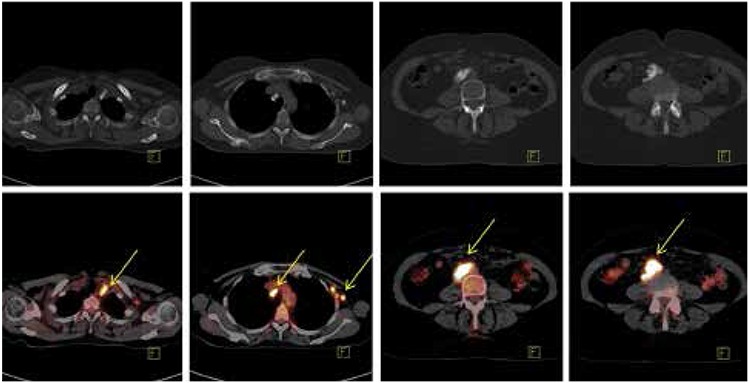
Calcified lymph nodes in the mediastinum and abdomen demonstrate high fludeoxyglucose uptake (arrows). Average SUVmax=8.78, highest SUVmax=12.7. Upper row: Calcifications as displayed by computed tomography (bone window). Lower row: Corresponding positron emission tomography/computed tomography images (soft tissue window)

**Figure 4:**
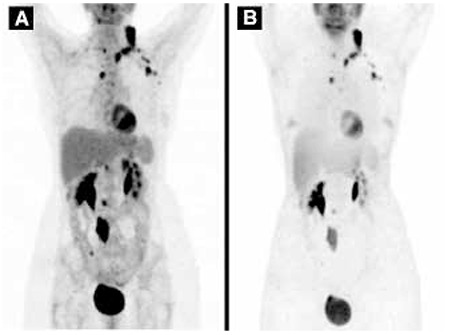
Both corrected (A) and uncorrected (B) images revealed high fludeoxyglucose uptake at the calcified lymph nodes, indicative of active disease

A 56 year-old woman had been treated 18 years ago for ovarian cancer. The patient underwent hysterectomy along with surgical removal of the fallopian tube and ovaries due to cancer of the right ovary. Histologic examination was consistent with well-differentiated serous papillary cystadenocarcinoma of the right ovary with scattered moderate differentiation, and metastatic invasion of the left ovary. Eighteen years later, the patient presented with a mass at the left supraclavicular space, corresponding to an enlarged lymph node. The fine needle aspiration biopsy (FNAB) of the enlarged supraclavicular lymph node revealed recurrence of the previously treated serous adenocarcinoma. Epithelial cells of cylindrical shape in papillary clusters, psammoma bodies and foamy histiocytes were observed. The patient underwent CT of the neck, thorax, upper and lower abdomen for evaluation of disease extent. Lymph nodes were detected at the neck, including the left supraclavicular area, the mediastinum, the left axilla, the right paraaortic region, the right common iliac and external iliac vessels, as well as the right retrocrural space. The lymph nodes were enlarged and displayed large amount of calcifications, while some of them were completely replaced by calcium depositions (Figure 1). The patient underwent chemotherapy with Taxol and Cisplatin (every 21 days) for three cycles, and Taxol and Carboplatin for the remaining three cycles, and was referred for evaluation of response to treatment. The CT revealed size reduction in the lymph nodes with progression of calcification at the previously described sites, with a density reaching up to ~900 HU in the calcified lymph nodes (Figure 2). The patient was referred for PET/CT examination for investigation of residual disease at the described lymph nodes and evaluation of treatment response six weeks after the last chemotherapy session. 50 minutes after intravenous administration of 362 MBq 18F-FDG, the PET/CT examination was performed by a Siemens Biograph LSO 16 sections device. Images were reconstructed at three levels, were corrected for attenuation and finally fused. Interpretation of PET/CT images was carried out by two experts (one nuclear medicine physician and one radiologist). Both corrected and uncorrected images were reviewed. Attenuation corrected FDG-PET/CT revealed hypermetabolism in all the described calcified lymph nodes, with a SUVmax ranging from 4.6 to 12.7, average SUVmax 8.78 (Figure 3). Uncorrected images also revealed active metabolic sites at all the described lymph nodes (Figure 4). The patient was referred for radiation treatment of the supraclavicular lymph node and was planned for close surveillance. On follow-up CT scans, additional calcifications were recognized in both the supraclavicular and the other lymph nodes. Follow-up fine needle aspiration of the still enlarged supraclavicular lymph node revealed metastasis from the known primary ovarian cancer, and the Magnetic Resonance Imaging revealed presence of another mass lesion of 5x3.5x3 cm size, which extended to the left axillary cavity. The patient underwent surgical removal of the supraclavicular lymph node and biopsy confirmed the diagnosis of metastasis and underlying multiple psammoma body deposits. After six months, the whole body CT revealed disease progression with multiple calcified lymph nodes as well as peritoneal implantation. A biopsy of the recently detected intra-tracheal mass confirmed the presence of new metastatic sites and moreover the presence of psammoma bodies. Based on the retrospective evaluation of patient’s medical history, these calcifications were interpreted as psammoma bodies formations, which was also consistent with the more indolent course of the patient’s disease.

## LITERATURE REVIEW AND DISCUSSION

Although not the leading female genital cancer, half of the deaths related to female genital system malignancies are attributed to ovarian cancer (1,2). Risk factors associated with ovarian cancer include menstrual and hormonal exposure events, expression of oncogenes and tumor suppressor genes, gonadotropins and steroid hormones, growth factors, age, demographic and environmental factors (2,8). Necrosis is usually detected in more aggressive forms of serous ovarian carcinoma. Calcification in these regions can be related to either necrosis or hemorrhage (3), and therefore it can be mistaken for necrotic tissue. Evaluation of those lymph nodes by PET may reveal potential hypermetabolism, indicative of active disease rather than necrosis. A suspicion of recurrence is usually raised based on serum tumor marker CA-125 elevation, although any radiologic lesion may not be apparent at that given time. However, its accuracy of predicting cancer is quite limited, as in 36-73% of ovarian cancer cases the serum CA-125 levels remain within normal values (1). Relapse becomes less likely as disease-free interval increases, especially after 5-year disease-free surveillance (9). Our case presented with recurrence after an 18-year disease-free period. Excluding second-look laparotomy, CT is usually the initial imaging procedure once recurrence is suspected. CT is also the first imaging procedure performed for evaluation of therapy response. However, its diagnostic accuracy is limited both for the differentiation of residual fibrous tissue from active disease and for the evaluation of lymph nodes with benign characteristics, such as those with normal size (1). The role of FDG-PET/CT in re-staging, in assessing therapy response, as well as in altering the patient’s management in suspicion of disease recurrence in ovarian cancer patients has been evaluated. The reported sensitivity, specificity, and accuracy rates in detecting suspected disease recurrence were reported as 74.2%, 90.9% and 82.6% for non-contrast enhanced CT (10). In another study, the accuracy of FDG-PET/CT for depicting recurrent tumor lesions according to anatomic localization was reported as 92% for the body, 96% for the chest, and 91% for the abdomen (11). Studies agree that PET/CT is more accurate than CT in detecting disease recurrence and reveal that it altered management decisions in over one third of patients (8,12). Lymph nodes with calcifications at CT are generally considered to be benign, with prior granulomatous disease being the most common etiology. It has been suggested that calcified mediastinal lymph nodes with high metabolic activity detected in NSCLC evaluation by PET/CT should not be necessarily considered as malignant, especially in countries where granulomatous diseases are endemic (5,13). Patterns of calcifications include amorphous, punctuate and linear types, and they can be detected either in the pre or post- therapy assessment, in primary and in metastatic lesions, either intra- or extra-abdominally (3,4,14,15). Calcification in ovaries is well described and is attributed to both neoplastic and non-neoplastic reasons (3,16). A few mechanisms have been proposed for the formation of calcifications such as hemorrhage, necrosis, mucinous degeneration, para-neoplastic syndrome and, most commonly in serous ovarian cancer, the formation of psammoma bodies. It is also suggested that tumor calcification in ovarian metastases is a dynamic process, independent of treatment effects, and therefore changes in calcification recognized on CT cannot be used as a marker of disease status (3). In our case, the calcified lymph nodes in the abdomen in the first pre-therapeutic CT examination were interpreted as probably benign, and attention was only paid to the supraclavicular enlarged lymph node. The CT performed after treatment revealed reduction in lymph node size and increase of calcified deposits with alterations of their deposition pattern from punctuate to complete replacement, which were interpreted as response to therapy and raised the question whether these axillary, mediastinal and abdominal calcified lymph nodes were also malignant. A FDG-PET was performed in order to differentiate active and inactive lesions, which revealed hypermetabolic activity in all calcified lesions with an average SUVmax of 8.78, evidence of active tissue. However, the mechanism of FDG uptake in calcified lymph nodes containing psammomas is yet unclear. Calcification, as occurs with a metallic and opaque material, may cause false positive findings in FDG-PET/CT. Artifacts provoked by i.v. contrast agents, chemotherapy catheters, and other dense and opaque materials when using CT-based attenuation correction protocols have been described in the literature (5). On the other hand, such findings may obscure active nearby disease. In order to avoid misinterpretation, both CT corrected and uncorrected images were reviewed, and hypermetabolism was detected by both methods at all calcified sites. Increase in FDG uptake after treatment due to flare phenomenon, causing false positive results, has also been described after Tamoxifen or Bevacizumab therapy (17,18). To our knowledge, such an effect has not been described for lymph node calcifications. Furthermore, our patient was treated with Taxane and Platinum and PET/CT examination took place six weeks after the last chemotherapy treatment. Psammoma bodies are composed of lamellated calcified structures organized in a concentric manner, and can be detected both in neoplastic and non-neoplastic conditions (3,14,19,20). They are reported to be present in 15-30% of patients with ovarian serous cystadenocarcinoma (14). They are more common in primary tumors, and rare in metastatic lesions. The mechanism by which psammoma bodies are formed remains unclear. They are reported to result from dystrophic calcification, from calcium accumulation in degenerated or necrotic cells, or from collagen calcification. They are also likely to represent a biologic process which leads to degeneration of cancer cells, with consequent death and delay of tumor growth (14,19,21,22). This last proposition suggests that their formation is the cause of the indolent character they provide to neoplastic cells. More investigation is required to elucidate their exact nature, their formation mechanism, and whether they are the result or the cause of retardation of tumor growth in ovarian cancer. Although the exact mechanism of psammoma body formation is yet unclear, they are known to be associated with increased apoptotic cell death, BRAF mutation, and normal TP53 function, all of which are more profound in low-grade ovarian serous adenocarcinoma (22,23,24). FDG uptake can occur through inflammatory processes secondary to these mechanisms. However, cases of psammocarcinomas with a more aggressive course have been described. Pyo et al. (25) demonstrated that psammoma deposits in papillary thyroid cancer are associated with multifocality and more extended disease. There are two distinct entities with regard to the deposition of psammomas in different carcinomas, carcinomas with psammoma bodies and psammocarcinomas. The later arise from the ovaries or peritoneum, and certain criteria must be fulfilled in order to characterize a carcinoma with psammoma bodies as psammocarcinomas (22). Cases reported in the literature include primary psammocarcinomas of the ovary or peritoneum, or psammoma deposits in benign and malignant conditions such as serous adenocarcinoma of the ovaries, thyroid, meningioma, pancreas and calcifications in metastatic lesions (3,6,14,15,16,20,21,22,25,26). Our case displays the presence of psammoma bodies in metastatic lymph nodes diagnosed during ovarian cancer relapse after 18 years. Apart from CT imaging, calcified axillary lymph nodes due to psammoma body deposition have been described in other imaging modalities, such as mammography (27) and Tc-99m-methyl diphosphonate bone scan (15).

## CONCLUSION

In conclusion, our case presentation has several remarkable characteristics. First, this case represents an ovarian serous carcinoma relapse after an 18-year disease-free interval. Second, it displays large calcified lymph nodes with increased density on CT images, which exhibit high FDG uptake in both attenuation corrected and uncorrected images, the last being evidence that increased FDG uptake is not an artifact. The formation of psammoma bodies could be suggested as a probable cause of calcification in this presented case. Similar to a previously described case, all sites of increased metabolic activity were considered as active tumor sites in the end.

## Ethics

Informed Consent: All authors have filled in the informed consent.

Peer-review: External and Internal peer-reviewed.

Financial Disclosure: The authors declared that this study has received no financial support.
